# The steady enhancement of the Australian Summer Monsoon in the last 200 years

**DOI:** 10.1038/s41598-017-16414-1

**Published:** 2017-11-23

**Authors:** David Gallego, Ricardo García-Herrera, Cristina Peña-Ortiz, Pedro Ribera

**Affiliations:** 1 Universidad Pablo de Olavide, Seville, Spain; 2 Universidad Complutense, Madrid, Spain; 3 Instituto de Geociencias UCM-CSIC, Madrid, Spain

## Abstract

A new bicentennial series of the Australian monsoon strength based on historical wind observations has allowed for the assessment of the variability of this system since the early 19th century. Our series covers a period in which the scarcity of meteorological observations in the area had precluded the evaluation of long-term climatic trends. Results indicate that the increase in precipitation over Northern Australia reported for the last 60 years is just a manifestation of a much longer lasting trend related to the strengthening of the Australian monsoon that has been occurring since at least 1816.

## Introduction

The Australian monsoon is the strongest monsoon in the Southern Hemisphere. Its seasonal cycle is initiated by a previous continental warming related to convergence at low levels in northern Australia^1^ that can be traced up to a month before the onset, which typically occurs in December. Once established and up to late February or early March, it is characterized by persistent westerlies below 700 hPa feeding the monsoon over a large area extending over the Java-Sumatra region, Java and Timor Sea and further eastward to Papua Nueva Guinea^2,3^. Historically, the strength of the Australian monsoon has been quantified by the summer precipitation at Darwin, which is available from the end of the 19th century. However, this series is not fully representative of the broad-scale Australian monsoon^4^ and dynamical indices based on wind averaged over the monsoon’s moisture source area are preferred^5–7^. Currently, the ‘AUStralian Monsoon Index’ (AUSMI)^4^ is the dynamical index most widely used. It is defined as the 850 hPa zonal wind averaged over the area bounded by [5S-15S, 110E-130E]. It relies on reanalysis data and is currently available from January 1948 onwards.

### Historical meteorological observations and climate indices

From the early days of ocean sailing, mariners kept written reports of the weather. By the late 18th century, these accounts were usually recorded in formal ships’ logbooks which have been preserved in historical archives^8,9^. Meteorological observations found in these logbooks contain first-hand and well-dated daily evidence of the weather that ships encountered along their route as an estimate of the state of the sea, information about precipitation (e.g. occurrence of rain or thunderstorm) and descriptors relative to the observed wind force and direction^8,9^. Among these data, wind measurement is the most important one from a climatic point of view. First, because due to its relevance for the sailing, it was taken on a daily or even sub-daily basis, supplying continuous coverage. Second, as the wind is directly related to the atmospheric dynamics, it has been possible to include these early meteorological observations into multiproxy reconstructions of the Sea Level Pressure in heavily navigated areas such as the North Atlantic^10–12^ and more recently to develop instrumental indices for the North Atlantic Oscillation^13^, the strength of northern Hemisphere monsoons^14,15^ or the El Niño Southern Oscillation^16,17^.

In recent years, and as a result of several international projects of data recovery^8,18,19^, the amount of available historical wind observations has increased significantly in other regions including the Indian and the South Pacific Oceans, opening the possibility for the development of long climatic indices for regions where no other *in-situ* meteorological observations are available. However, as pointed out by Gallego *et al*.^20^, due to the characteristics of this source of data, the direct use of the historical wind vector (force and direction) to compute climate indices can introduce non-climatic biases. In most of the cases, prior to the mid-20th century, wind force on sailing ships was not measured with instruments but estimated and codified as “wind descriptors”. These could be annotated in non-standard style even after the implementation of the Beaufort scale during the 19th Century (the precise date for the adoption of this scale depends on the country). Historical wind force estimates are therefore subject to the interpretation of the terms used to describe the wind strength prior to their numerical conversion^21^. In contrast, wind direction observations do not suffer such limitation. Wind direction was measured with a 16 or 32-point compass with respect to the magnetic north, enabling each measurement to be referred to the true north using the magnetic variation. Wind direction does not require subjective judgements or even re-scaling to modern standards. In fact, this variable can be considered a truly instrumental observation, even for the oldest records^13^ and in consequence, climate indices based on this variable can be regarded as instrumental ones.

### The Australian Monsoon Directional Index

**Figure 1:**
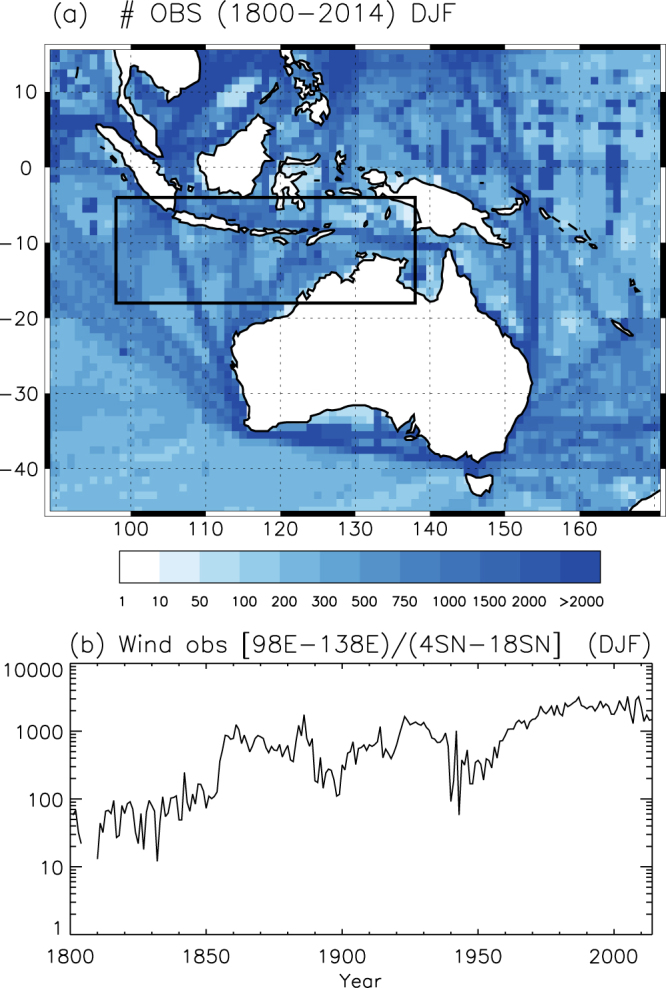
(**a**) Number of wind direction observations in a 1° × 1° grid during the austral summer (DJF) for the 1800–2014 period available in ICOADS 3.0. Black rectangle indicates the area selected to compute the AMDI. (**b**) Time evolution of the cumulative number of wind direction observations inside the [4°S-18°S, 98°E-138°E] domain (December-February). Map was created using IDL Version 6.3 (http://www.harrisgeospatial.com/ProductsandTechnology/Software/IDL.aspx).

Currently, most of the early wind direction measurements over the oceans retrieved from ship’s logbooks as a result of recovery projects have been incorporated in the International Comprehensive Ocean-Atmosphere Data Set (ICOADS) database^22^, whose most recent release (v3.0) holds over 456 million individual marine reports covering the period 1662–2014. A number of these observations concern voyages between Europe and Asia or Australia following routes that, in most cases, crossed the area directly affected by the Australian Monsoon. Based on these data, our hypothesis is that the frequency of days with prevalent westward wind in a certain geographical domain over Northern Australia is representative of the changes in zonal wind speed at the 850 hPa level measured by the AUSMI. As a consequence, it would capture the variability of the moisture transport associated with this monsoon. Under this assumption, we have defined the Australian Monsoon Directional Index (AMDI) as the percentage of days per month inside the domain [4S-18S, 98E-138E] (see Fig. 1) with prevailing surface wind blowing from the west according to the data stored in the ICOADS database. Here we refer to days with ‘prevailing wind blowing from the west’ as those when at least 53% of the daily reports within the specified domain correspond to wind observations from that direction +/−45 degrees (i.e. between 225° and 315° from the true north). Both the area and the percentage were determined in a calibration process designed to optimize the correlation between the AMDI and the AUSMI for their common period 1948–2014. The correlation reaches r = +0.69 and r = +0.74 (p < 0.01) for January and February (the core of the Australian monsoon season) and r=+0.71 (p < 0.01) for December. Correlation values are rather stable independently of the period considered. Nonetheless they tend to be larger over more recent periods, for which the AUSMI is considered more accurate and ICOADS contains a larger number of observations. For example, the AUSMI-AMDI correlation during the post satellite 1979–2014 period reaches r = +0.75 and r = +0.86 (p < 0.01) for January and February and r = +0.81 for December (p < 0.01).

**Figure 2:**
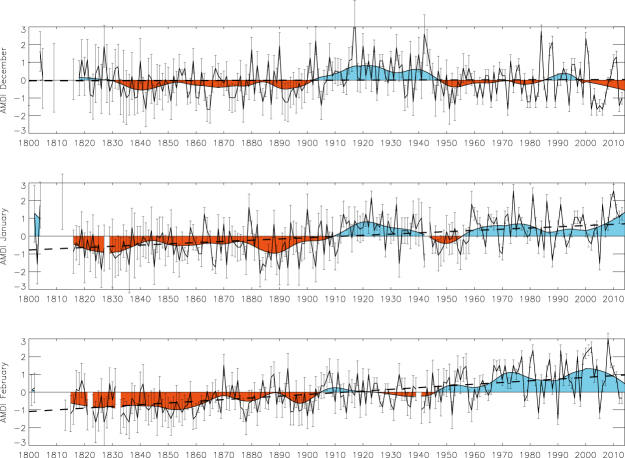
Standardized AMDI for December, January and February between 1800 and 2014. Error bars indicate the expected standard deviation based on the number of observations available each year in ICOADS 3.0. Shaded curve is computed as a robust locally weighted regression with a 31-year window^19^. Dashed lines indicate the linear fit (1800–2014). Linear trends for December, January and February are 0.00, +0.69 (p < 0.01) and +0.96 (p < 0.01) standard deviations per century respectively.

**Figure 3:**
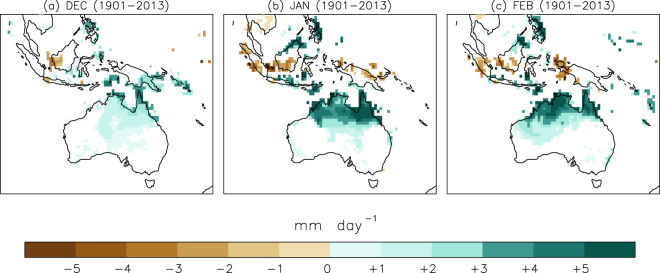
Precipitation differences between years with AMDI over +/−1 standard deviation for the 1900–2013 period as seen from GPCCv7 data. Only areas with precipitation differences statistically significant (p < 0.05) based on a 1,000 trial Monte Carlo test are represented. Maps were created using IDL Version 6.3 (http://www.harrisgeospatial.com/ProductsandTechnology/Software/IDL.aspx).

**Figure 4:**
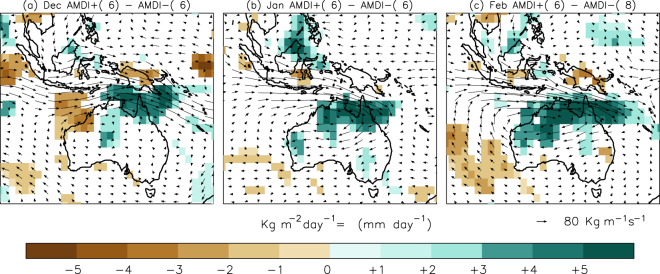
1000–700 hPa vertically integrated moisture transport (arrows, scale at the lower-right corner) and moisture convergence (shaded areas) differences between AMDI +/−1 standard deviations years for the 1979–2014 period, using NCEP/NCAR reanalysis data. Only significant moisture convergence differences (p < 0.05) based on a 1,000 trial Monte Carlo test are represented. The number of positive and negative AMDI cases used to compute the anomalies is indicated in brackets. Maps were created using IDL Version 6.3 (http://www.harrisgeospatial.com/ProductsandTechnology/Software/IDL.aspx).

Figure 1a shows the selected area (black rectangle) and the spatial distribution of the number of ICOADS observations for the austral summer, evidencing the busiest shipping lanes. Figure 1b displays the evolution of the number of wind direction observations during the austral summer (DJF) inside the selected area. Up to 1850, this number is usually under 100 observations per season. From 1850 on the number increases to typical values of several hundred observations per season, with the exception of some data shortage during the last decade of the 19th century and the Second World War period. From 1970 onwards there are always more than 1000 observations per season inside the area. We required a minimum of 10 days with observations per month to compute the AMDI, otherwise the index was tagged as missing (see the Methods section for an analysis of the expected dispersion associated to the number of observations). Figure 2 illustrates the resulting standardized AMDI for December, January and February. The smoothed curve is computed as a locally weighted regression robust against the presence of outliers with a 31-year window width^23^. Error bars indicate the uncertainty estimated from the expected standard deviation of the ADMI value based on the number of wind measurements available each year (a lower number of observations implies larger uncertainties, see the Methods section). To evaluate rainfall changes linked to the AMDI we computed the difference between precipitation during months with AMDI above/below one standard deviation with respect to its average value over the period 1901–2013, which is covered by the Global Precipitation Climatology Centre v7 dataset^24^. The result (Fig. 3) proves a clear monsoonal structure with significant changes in precipitation extending over all tropical Australia and spreading southward into parts of the semi-arid terrains in Western Australia and the Northern Territory. The largest anomalies reach values above 5 mm·day^−1^ in January and February over the Kimberley region in Western Australia, Arnhem Land in the Northern Territory and the Cape York Peninsula in Queensland. The corresponding moisture transport based on NCEP/NCAR reanalysis data^25^ is shown in Fig. 4 through the 1000–700 hPa vertically integrated moisture transport and its related convergence for years with AMDI above/below one standard deviation. We limited this analysis to the 1979–2014 period due to the large uncertainties in the vertical distribution of the specific humidity in the NCEP/NCAR reanalysis prior to the satellite era in the southern hemisphere. The agreement between Figs. 3 and 4 is remarkable both in the areas affected and in the precipitation/convergence anomalies. This indicates that, even though the AMDI does not contain wind speed information, large values are synchronous to anomalous westerly moisture transport from the Indian Ocean into the Timor Sea and the Pacific, increasing the monsoonal precipitation in 3 to 5 mm·day^−1^ for most of northern Australia when compared with low AMDI values.

As described in the introduction, the correlation between dynamical indices for the Australian monsoon, like the AUSMI^4^, and the precipitation at Darwin (January to February) is not very high (r = +0.53, p < 0.01 for the 1949–2010 period). In spite of this, it is worthwhile to make use of this exceptionally long record of precipitation, which starts in 1870, to assess the AMDI stability. We have also used this series to examine the performance of AUSMI-like dynamical indices computed from data from the NOAA-CIRES Twentieth Century Reanalysis Project version 2c (20CR-V2c), covering the 1851–2012 period^26^ and the ECMWF Twentieth Century Reanalysis (ERA-20c), available between 1901 and 2010^27^. Table 1 shows that the correlation between the AMDI and the Darwin’s precipitation is rather constant independently of the considered period, showing values around r = +0.40 (p < 0.01), which are comparable to those found for the ERA20c or 20CR-V2c. In fact, as both 20CR-V2c and ERA-20c incorporate ICOADS data^26,27^, it is not surprising that correlations between monsoonal precipitation and dynamical indices based on historical reanalysis produce similar values. However, the simplicity of the AMDI allows to compute it since 1816 with only minor gaps. Additionally, it can be considered a direct instrumental series, not affected by possible biases in the process of data assimilation. As such, the AMDI provides the first direct observational evidence of the continuous increase in the Australian monsoon since the early 19th century.

**Table 1:** Correlation coefficients between the seasonal precipitation at Darwin (December to February) and: The zonal wind at 850 hPa averaged over the [5S-15S, 110E-130E] for the 20CR-V2 and the ERA-20c reanalysis, the AUSMI^4^ and the AMDI. All values are statistically significant at p < 0.01.

Period	20CR-V2c	ERA-20c	AUSMI	AMDI
1870–2010	0.40	—	—	0.36
1901–2010	0.45	0.42	—	0.42
1949–2010	0.51	0.42	0.53	0.41

### Observed increase in the Australian Monsoon strength

The AMDI evolution clearly shows that the core of the monsoon season (January and February) has suffered a noticeable increase in the frequency of the westerlies since the 1950’s and that the frequency of westerlies in northern Australia prior to 1900 was noticeably lower than today. Although the uncertainty of each individual AMDI value prior to the 1930’s is rather large due to the relatively small number of observations, the limited evidence previously available is in line with this result^28^. The increase in the summer rainfall across north Australia during the 20th Century has been previously documented for the 20th century^29^ and specifically from the 1950’s onward^30–32^. This has been consistently related to intensification of the total cloud amount and the extension of monsoon rainforest^33,34^. However, there is still considerable uncertainty about the significance of this observed trend at longer timescales. Proxy records related to the Australian monsoon are extremely limited over Northern Australia^35^. This is largely due to the difficulties in identifying suitable tropical tree species with annual rings allowing a precise crossdating^36^ or in isolating robust climatic signals in corals sampled at the Great Barrier Reef^37^. Notwithstanding, most of the research on the Australian monsoon history indicates that this system could have been rather variable through the Holocene^38–42^. Unfortunately, high-resolution proxies covering the last few centuries at the annual scale are really scarce. The few high resolution proxy reconstructions of monsoonal rainfall in Australia suggest that the 19th century could indeed have been characterized by a weak monsoon. Lough^37^ reported a likely tendency towards lower than present precipitation between 1760 and 1850, by using quantitative measurements of coral luminescence as a summer precipitation proxy in North West Queensland. However the author pointed out that the results were not conclusive. More recently, O’Donnell *et al*.^43^, using tree ring chronologies, proposed that the recent increase in summer-autumn precipitation recorded at northwest Australia probably did not have precedence in the last two centuries, although their analysis is not strictly representative of the tropical monsoonal regime but rather of the semi-arid territories southward of 20°S. The AMDI record is able to put these pieces into a context of continuous monsoon strengthening.

Current limitations of climate models’ reproducing the present-day monsoonal regimes limit the confidence in their future projections. Recently, Kitoh *et al*.^28^ found a significant model spread for the case of the Australian monsoon in 29 models included in the CMIP5 framework. Although it is believed that the Australian monsoon will intensify under increasing Greenhouse gases concentrations^44^, modelled precipitation for Northern Australia is still highly dependent upon the model used^45,46^. Recently, it has been pointed out that a large part of this uncertainty likely lies in observational limitations in Australia rather than in poorly represented processes in models^47^. In this context, the AMDI index is relevant to improve the knowledge of the long term Australian monsoon variability and the Northern Australian precipitation. It provides the first instrumental record of the Australian monsoon strength covering two centuries and it shows that, embedded into large interannual variability, the intensity of the Australian monsoon has been steadily increasing at least since 1816.

## Methods

### Estimation of the AMDI uncertainty

The inherent spatial variability of the wind inside the area selected to compute the AMDI and the finite number of available measurements in a given month is translated as dispersion or ‘uncertainty’ in a particular realization of the index. To estimate this uncertainty we considered the 1971–2010 period (in order to have a large enough pool of observations). For each of the summer months, considering the whole 40 year period, we computed 40,000 (1,000 per year) ‘degraded’ AMDI indices constructed from N randomly chosen wind observations inside the [4S-18S, 98E-138E] domain, with N ranging from 10 to 500. For each value of N, the 40,000 degraded AMDIs are expected to be different because they are constructed from a different set of observations. The standard deviation of these series as a function of N for December, January and February can be interpreted as the expected uncertainty of a particular realization of the AMDI computed from a given number of measurements. As expected, the largest standard deviations are found for the indices computed from a lower number of observations (N = 10, standard deviation around 17%). This value rapidly decreases as N increases. For N = 50 the standard deviation is well below 10%, while for N over 400 the standard deviation is almost stable and around 6% (January and February) and 5% (December). The fact that the standard deviation does not tend to cero as N increases reflects the intrinsic spatial variability of the wind inside the selected region (which spans around 7,000,000 km^2^). The error bars of each AMDI value displayed in Fig. 2 are thus exclusively based on the number of observations available each year. For the cases with N > 500, the value for N = 500 has been used. It must be pointed out that this dispersion measure is purely empirical. Thus, it should only be interpreted as the estimated standard deviation of an AMDI value computed from a number N of wind direction measurements and not as a confidence interval in a statistical sense.

### Data Availability

All data used in this paper are freely available at http://www.esrl.noaa.gov/psd/ (NCEP, GPCC, 20CR-V2), http://apps.ecmwf.int/datasets/data/era20c-daily/ (ERA-20c), https://rda.ucar.edu/datasets/ds548.0/ (ICOADS), and http://www.bom.gov.au/climate/data/stations/ (Darwin precipitation).
